# Salvinorin A inhibits ovalbumin‐stimulated allergic rhinitis and RBL‐2H3 cells degranulation

**DOI:** 10.1002/2211-5463.13219

**Published:** 2021-06-25

**Authors:** Qiyang Shou, Tao Tan, Faying Xu

**Affiliations:** ^1^ The Second Affiliated Hospital Zhejiang University of Chinese Medicine Hangzhou China; ^2^ Internal Medicine Department Zhejiang Provincial General Team Hospital of the Chinese People's Armed Police Force Hangzhou China; ^3^ School of Medical Imaging Hangzhou Medical College China

**Keywords:** allergic rhinitis, inflammation, mast cells, ovalbumin, salvinorin A

## Abstract

Allergic rhinitis (AR) is a long‐term noncommunicable inflammatory disease of the nasal mucosa mediated by immunoglobulin E and is mainly caused by exposure of genetically susceptible individuals to environmental allergens. Mast cells contribute to the pathogenesis of allergic and nonallergic inflammatory diseases. Salvinorin A has been previously shown to inhibit leukotriene production and mast cell degranulation to suppress airway hyperresponsiveness caused by sensitization; thus, we hypothesized that salvinorin A has an anti‐AR effect. We tested this hypothesis using monoclonal anti‐2,4,6‐dinitrophenyl immunoglobulin E/human serum albumin‐induced rat basophilic leukemia cells (RBL‐2H3 cells) and ovalbumin (OVA)‐induced AR in mice as *in vivo* and *in vitro* AR models, respectively. The expression levels of histamine, β‐hexosaminidase, interleukin‐4 and tumor necrosis factor‐α were decreased by salvinorin A *in vitro*. Granule release and F‐actin organization were also suppressed by salvinorin A. Furthermore, salvinorin A inhibited OVA‐induced features of AR in mice, including nasal rubbing and sneezing, as well as increased OVA‐specific immunoglobulin E, histamine, tumor necrosis factor‐α and interleukin‐4 levels. In addition, salvinorin A decreased the phosphorylation of phosphoinositide 3‐kinase/Akt *in vitro* and *in* *vivo*. Our work suggests that salvinorin A suppresses AR caused by sensitization by inhibiting the inflammatory responses of mast cells; thus, salvinorin A may have potential for treatment of AR.

## Graphical abstract

Salvinorin A inhibited mast cell degranulation and ovalbumin‐induced allergic rhinitis (AR) in mice. In addition, salvinorin A down‐regulated the phosphorylation of phosphoinositide3‐kinase/Akt *in vitro* and *in* *vivo*. Overall, our work demonstrated that salvinorin A suppresses AR caused by sensitization through inhibition of inflammatory responses of mast cells, and salvinorin A may be a promising candidate for new therapeutic approaches to AR.

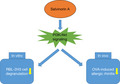

**Abbreviations**
Akt: protein kinase B
AR: allergic rhinitis
DNP‐IgE/HSA: anti‐2,4,6‐dinitrophenyl‐immunoglobulin E/human serum albumin
IgE: immunoglobulin E
IL‐4: interleukin‐4
IL‐6: interleukin‐6
LT: leukotriene
OVA: ovalbumin
PI3K: phosphoinositide‐3‐kinase
SEM: standard error of the mean
TNF‐α: tumor necrosis factor‐α

Allergic rhinitis (AR) is a long‐term noncommunicable inflammatory disease of the nasal mucosa mediated by IgE and is mainly caused by exposure of genetically susceptible individuals to environmental allergens. AR is a global health problem that currently affects up to 40% of the general population and is characterized by symptoms that vary in severity and duration, including congestion, itching, watery nose and sneezing [1, 2, 3].

Studies have shown that mast cells contribute to the pathogenesis of allergic and nonallergic inflammatory diseases, such as AR, allergic conjunctivitis and chronic sinusitis [4, 5, 6]. Mast cells are often abundant in areas of the body that are in contact with the outside world, such as the skin, airways, gastrointestinal tract and other tissues that are exposed to the environment; therefore, mast cells are not only major effector cells but are also known as ‘key sentinel cells’ in the natural immune system [7, 8]. Mast cells are characterized by a combination of immunoglobulin E (IgE) and the high‐affinity IgE receptor (FcεRI) on the cell membrane surface to activate and induce mast cell threshing [9]. During mast cell degranulation, members of the phosphatidylinositol‐3‐kinase (PI3K) family play an important role in mast cell‐induced mediators [10].

Salvinorin A is a miraculous diterpenoid isolated and purified from the leaves of psychedelic sage and has been shown to be the main active ingredient in psychedelic sage [11]. In the 1990s, Siebert [12] demonstrated that salvinorin A could affect the mind. Salvinorin A is a highly selective kappa agonist and is more efficient than the classic kappa agonists U69593 and U50488 [13]. In addition, the antiallergic effect of salvinorin A has already been published in previous work [14]. They showed that salvinorin A inhibited airway hyperreactivity, and this effect was sustained by inhibition of mast cell degranulation/leukotriene (LT) production. However, the underlying mechanisms of the inhibition effects of salvinorin A acted in the activation of mast cell, and AR remains unknown. Therefore, we speculated that salvinorin A has a protective role against AR. Herein, we explored the role of salvinorin A in ovalbumin (OVA)‐triggered AR mice and anti‐2,4,6‐dinitrophenyl‐immunoglobulin E (IgE)/human serum albumin (DNP‐IgE/HSA)‐stimulated rat basophil cells (RBL‐2H3 cells) and studied the underlying mechanisms using various biological methods.

## Materials and methods

### Cell culture and IgE‐mediated mast cell activation

The RBL‐2H3 cell line was purchased from the Type Cell Culture Collection of the Chinese Academy of Science (Shanghai, China). Cells were grown in culture dishes filled with minimum essential medium (HyClone Laboratories, Logan, UT, USA) supplemented with 15% FBS (Gibco, Carlsbad, CA, USA) and 100 μg·mL^−1^ streptomycin/penicillin. The cells were placed in an incubator with 1.5 mg·mL^−1^ sodium bicarbonate, 110 μg·mL^−1^ sodium pyruvate and 5% CO_2_ at 37 °C. After incubation in a 48‐well plate for 12 h, cells were treated with 100 ng·mL^−1^ DNP‐IgE (Sigma‐Aldrich, MO, USA) for another 12 h and pretreated with salvinorin A (purity: 99% by HPLC; Sigma‐Aldrich) for 1 h. The earlier treated cells were stimulated with 250 ng·mL^−1^ DNP‐HSA (Biosearch, Petaluma, CA, USA) for 12 h. There were three groups: the control group, DNP‐IgE/HSA group and DNP‐IgE/HSA + salvinorin A group.

### Cell viability

Salvinorin A at 0–500 μm was used to treat RBL‐2H3 cells. MTT solution was added to the cells for 4 h. After the cells fully responded, they were detected with a microplate reader (Thermo Fisher Scientific, Waltham, MA, USA) at an absorbance intensity of 450 nm (*A*_450 nm_), and the results were recorded and analyzed.

### Assessment of histamine and β‐hexosaminidase

Supernatants were centrifuged for 10 min at 4 °C, and then 50 μL of supernatant was collected into a new 1.5‐mL tube, thoroughly mixed with 50 μL of substrate (1 mm p‐nitrophenyl‐*N*‐acetyl‐β‐d‐glucosaminide in 0.1 m sodium citrate buffer) and incubated at 37 °C for 1.5 h. *A*_405_ _nm_ was measured for β‐hexosaminidase detection. ELISA kits (Elabscience Biotechnology Co., Ltd., Wuhan, China) were used to evaluate the release of histamine.

### Interleukin‐6 and tumor necrosis factor‐α assessment

Cells were seeded in six‐well plates, cultured in a 37 °C incubator overnight to 80% density, and then treated as described earlier. Then, the supernatants were collected to detect the expression levels of interleukin‐6 (IL‐6) and tumor necrosis factor‐α (TNF‐α) with ELISA kits (Elabscience Biotechnology Co., Ltd.).

### Toluidine blue staining

Cells that were treated as described earlier were rinsed with PBS for 2 min, fixed with 4% paraformaldehyde at room temperature for 30 min, washed with PBS, impregnated with 1% toluidine blue for 2 h, redyed with hematoxylin and washed with double‐distilled H_2_O to remove the excess dye. Then, the cells were gradient dehydrated in different concentrations of alcohol from 70% to 95% and cleared with xylene. After sealing with neutral resin, images were acquired with a microscope and analyzed.

### F‐actin microfilament staining

The sensitized cells were pretreated with salvinorin A, the medium was discarded after removal from the incubator and the residual medium was washed away with PBS. The cells were fixed with 4% paraformaldehyde and treated with 0.1% Triton X‐100/PBS for 5 min to disrupt the membranes. Alexa Fluor 488‐phalloidin diluted in 1% BSA was used to stain the cells for 30 min. Eventually, F‐actin microfilaments were detected with a Leica DM2500 microscope (Leica Microsystems, CMS GmbH, Wetzlar, Germany) at an excitation wavelength of 490 nm and an emission wavelength of 520 nm.

### Western blot

The protein samples were taken from cells and tissues. Then protein lysates were fully lysed for 30 min in ice and centrifuged at 12 000 ***g*** for 5 min at 4 °C. After discarding the precipitate, the supernatant was collected and diluted with 5× loading buffer in a ratio of 1 : 4, then heated for 5 min at 100 °C for fully protein denaturation. For western blot, the samples were electrophoretically separated on SDS/PAGE, then electrotransferred to polyvinylidene difluoride membranes. After blocking in western blocking fluid for 2 h at a shaker and washing the residual liquid, the membranes were incubated well at 4 °C for overnight with primary antibody, which was prediluted with western primary antibody dilution buffer. After washing the membranes in 1× TBST three times, the corresponding secondary antibody conjugated with goat anti‐rabbit or goat anti‐mouse HRP was incubated for 2 h. After washing the membranes three times, detection of proteins was performed with BeyoECL Plus kit (Shanghai Haling Biological Technology Co., Ltd, Shanghai, China).

### Animals and OVA‐induced AR in mice

Female BALB/c mice (25 ± 2 g) were procured from the Shanghai Slake Laboratory Animal Co., Ltd (Shanghai, China) and subjected to a stable environment with a relative humidity of 50–60% and a temperature of 23 ± 2 °C. In addition, mice were housed under normal laboratory conditions with a 12‐h light–dark cycle. All the procedures on mice in this study were performed in rigorous compliance with the National Institutes of Health *Guide for the Care and Use of Laboratory Animals* and were approved by the Institutional Animal Care and Use Committee of The Second Affiliated Hospital of Zhejiang University of Chinese Medicine.

Mice were injected intraperitoneally with 2 mg of aluminum hydroxide containing OVA (50 μg) every 2 days for 14 days. Then, sensitized mice were obtained by injecting 10 μL of 10% OVA into the bilateral nasal cavities for 10 consecutive days. The Salvinorin A (SA) treatment group received an intraperitoneal injection of 10 or 20 mg·kg^−1^ SA from days 15 to 24 before intranasal OVA treatment. The control group received only saline injections. The amount of nasal rubbing and sneezing was measured for 10 min after intranasal OVA stimulation. Twenty‐four hours after the last intranasal stimulation, we obtained the serum of the mice, and the expression levels of OVA‐specific IgE, histamine, interleukin‐4 (IL‐4) and TNF‐α were evaluated via ELISAs, and the nasal mucosa tissues were obtained for western blot.

### Statistical analyses

Data were analyzed using graphpad prism 7 (GraphPad Software Inc., CA, USA) and are expressed as the mean ± standard error of the mean (SEM). Statistical significance was determined via one‐way ANOVA, followed by Bonferroni’s *post hoc* test. *P* < 0.05 was regarded as significant.

## Results

### Salvinorin A had an inhibitory effect on RBL‐2H3 degranulation

**Fig. 1:**
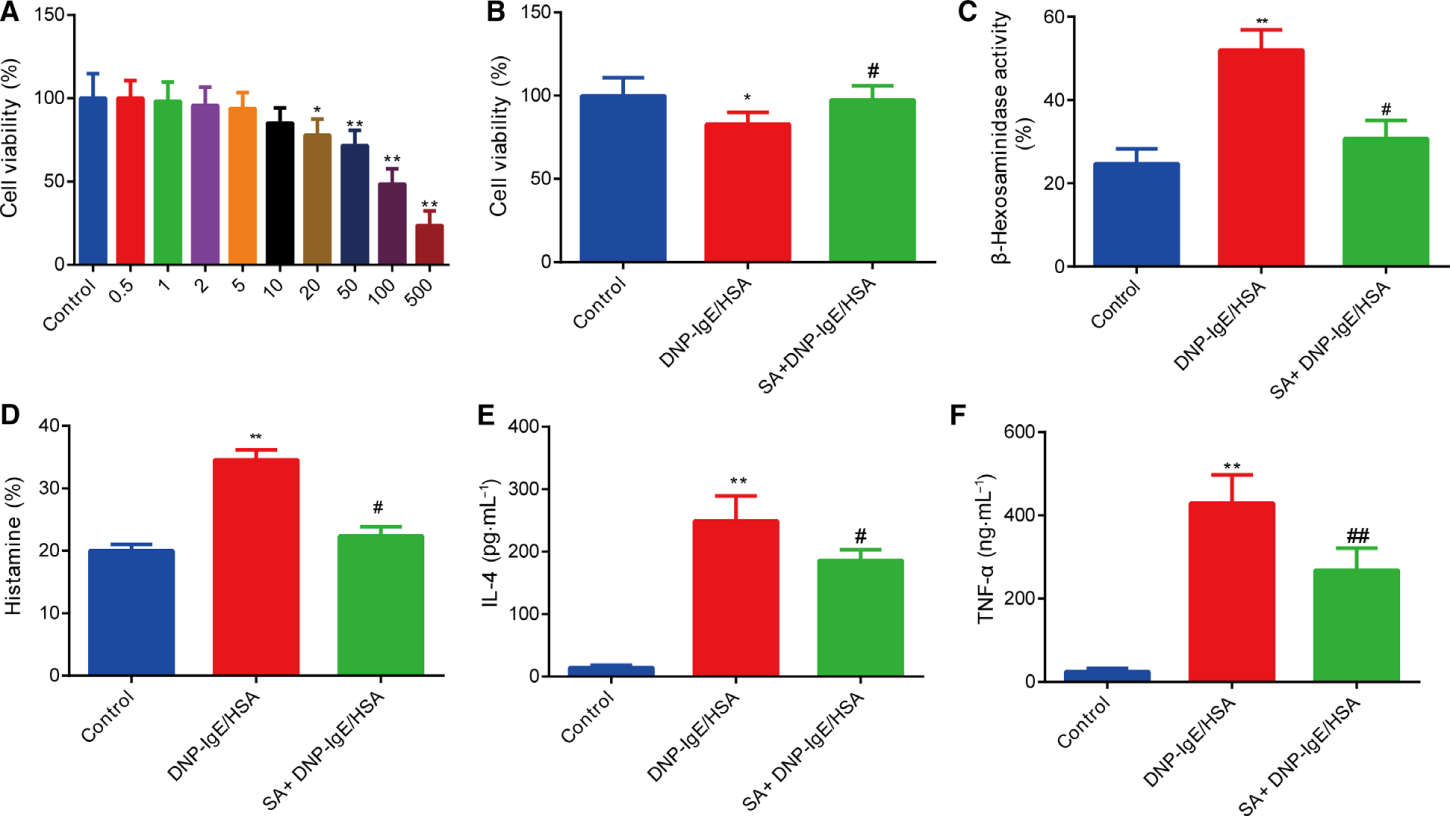
Salvinorin A had an inhibitory effect on RBL‐2H3 degranulation. (A) Effects of various concentrations of salvinorin A on the viability of RBL‐2H3 cells determined using MTT assays. (B) Salvinorin A improved DNP‐IgE/HSA‐stimulated RBL‐2H3 cell viability. (C) Salvinorin A inhibited the level of β‐hexosaminidase in DNP‐IgE/HSA‐stimulated RBL‐2H3 cells. (D) Salvinorin A inhibited the level of histamine in DNP‐IgE/HSA‐stimulated RBL‐2H3 cells. (E) Salvinorin A suppressed the level of IL‐4 in DNP‐IgE/HSA‐stimulated RBL‐2H3 cells. (F) Salvinorin A suppressed the level of TNF‐α in DNP‐IgE/HSA‐stimulated RBL‐2H3 cells. All data from three independent experiments are expressed as the mean ± SEM. *P < 0.05, **P < 0.01, in comparison with the control group; ^#^ *P* < 0.05, ^##^ *P* < 0.001, in comparison with the DNP‐IgE/HSA group. The difference between different groups was analyzed by one‐way ANOVA, followed by Bonferroni’s *post hoc* test.

MTT assays were conducted to examine the effect of salvinorin A on cell viability and to ensure that the reduction in mast cell granule levels could not be attributed to cell mortality. Salvinorin A (0–10 μm) had no apparent impact on the RBL‐2H3 cell survival rate (Fig. 1A). We selected 5 μm salvinorin A for subsequent experiments. Salvinorin A improved the survival of DNP‐IgE/HSA‐treated RBL‐2H3 cells (Fig. 1B). To learn more about the allergic inhibitory effect of salvinorin A on mast cell degranulation, we measured the levels of β‐hexosaminidase and histamine, which were two indicators of degranulation. Compared with the DNP‐IgE/HSA group, the levels of β‐hexosaminidase and histamine were lower in salvinorin A + DNP‐IgE/HSA‐treated RBL‐2H3 cells (Fig. 1C,D). TNF‐α and IL‐4, released during mast cell activation, are major key cytokines that promote inflammation. Thus, we determined the influence of salvinorin A on the expression of IL‐4 and TNF‐α in RBL‐2H3 cells. In our study, salvinorin A obviously suppressed the overexpression of IL‐4 and TNF‐α (Fig. 1E,F).

### Salvinorin A suppressed degranulation and F‐actin rearrangement in RBL‐2H3 cells

**Fig. 2:**
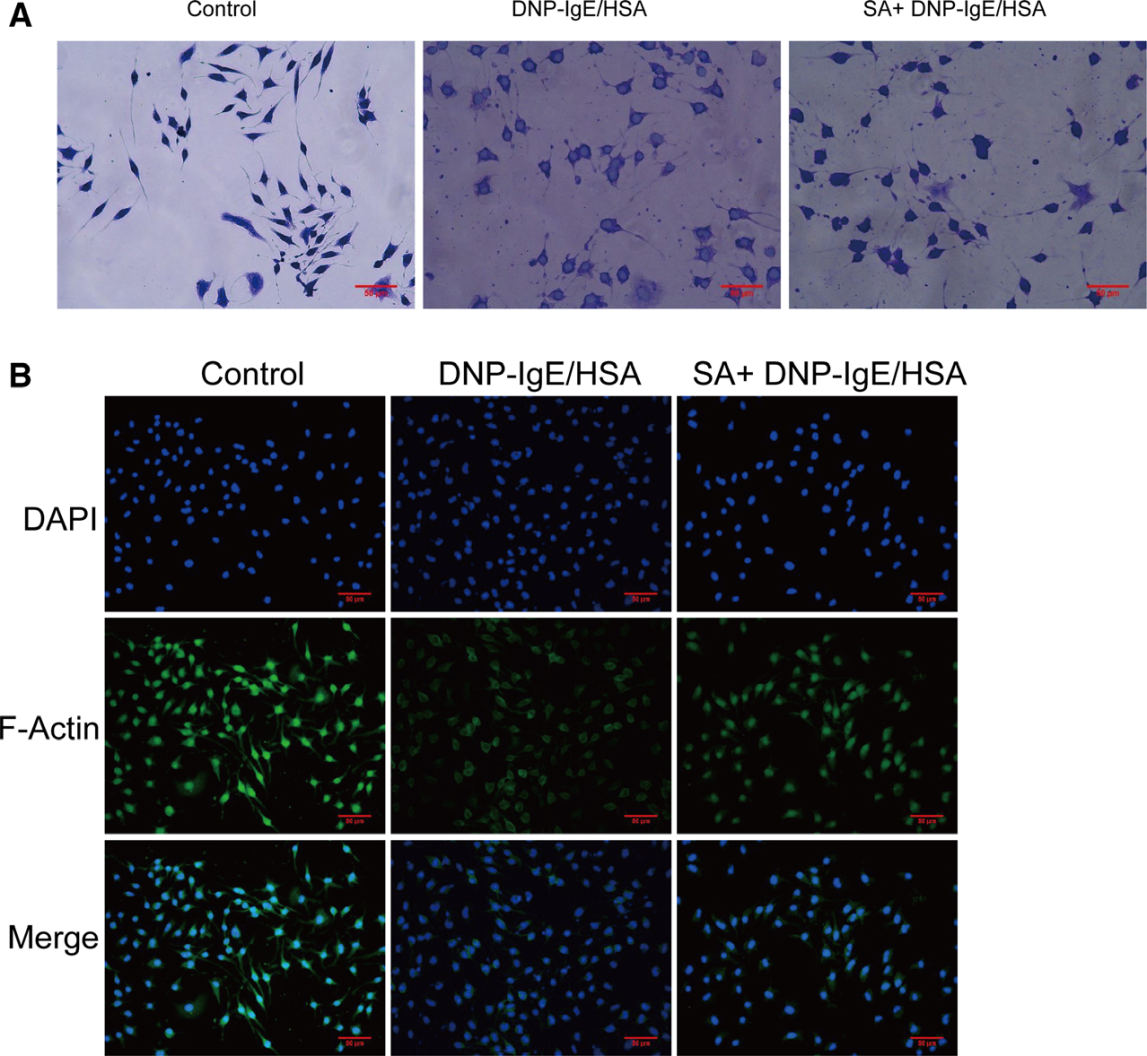
Effect of salvinorin A on granule release and F‐actin rearrangement in DNP‐IgE/HSA‐stimulated RBL‐2H3 cells. (A) Effects of coptisine on toluidine blue staining in DNP‐IgE/HSA‐sensitized cells. (B) Effects of coptisine on Alexa Fluor 488 phalloidin staining in DNP‐IgE/HSA‐sensitized cells. Scale bars: 50 μm.

The effect of salvinorin A on toluidine blue dye staining in RBL‐2H3 cells was investigated to visualize the release of granules. Normal RBL‐2H3 cells have an elongated morphology with purple granules inside. DNP‐IgE/HSA‐stimulated RBL‐2H3 cells were irregular in morphology and released purple granules, and salvinorin A treatment significantly suppressed morphological changes and degranulation (Fig. 2A). Changes in F‐actin in DNP‐IgE/HSA‐sensitized RBL‐2H3 cells were observed via Alexa Fluor 488‐phalloidin staining. Regular RBL‐2H3 cells were fusiform, and F‐actin was uniformly distributed around the cells. DNP‐IgE/HSA‐sensitized RBL‐2H3 cells were elliptical due to disintegration of the F‐actin cytoskeleton, and pretreatment with salvinorin A inhibited the morphological changes and disintegration of the F‐actin cytoskeleton (Fig. 2B).

### Salvinorin A repressed PI3K/Akt signaling in RBL‐2H3 cells

**Fig. 3:**
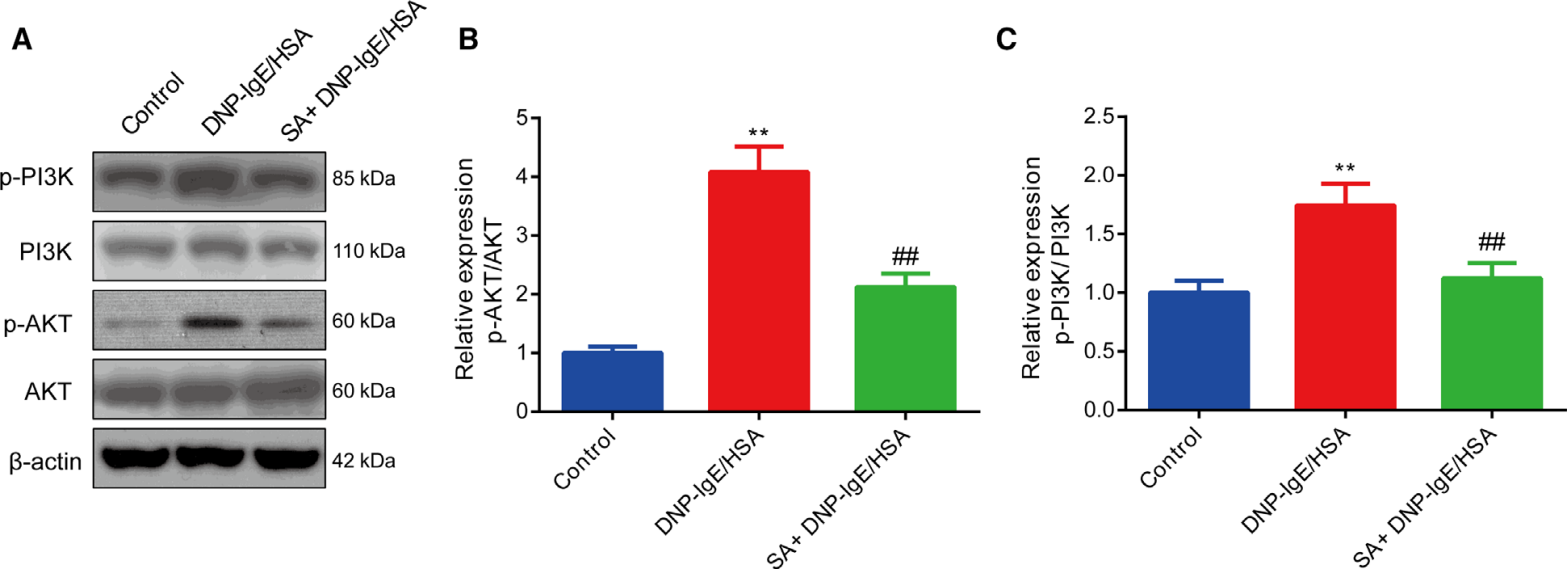
Salvinorin A repressed PI3K/Akt signaling in RBL‐2H3 cells. (A) The phosphorylation levels of PI3K (B) and Akt (C) in RBL‐2H3 cells (*in vitro*) were evaluated by western blotting. **P < 0.01, in comparison with the control group; ^##^ *P* < 0.001, in comparison with the DNP‐IgE/HSA group. All data from three independent experiments are expressed as the mean ± SEM. The difference between different groups was analyzed by one‐way ANOVA, followed by Bonferroni’s *post hoc* test.

To further study the mechanism underlying the inhibitory effects of salvinorin A on the activation of mast cells, we performed western blotting assays to evaluate the expression levels of PI3K, p‐PI3K, Akt (also named protein kinase B) and p‐Akt. The results showed that PI3K and Akt phosphorylation was significantly up‐regulated in the DNP‐IgE/HSA group. In contrast, in the group treated with salvinorin A, the expression of all the earlier proteins was reduced (Fig. 3A–C).

### Salvinorin A inhibited OVA‐induced AR in mice

**Fig. 4:**
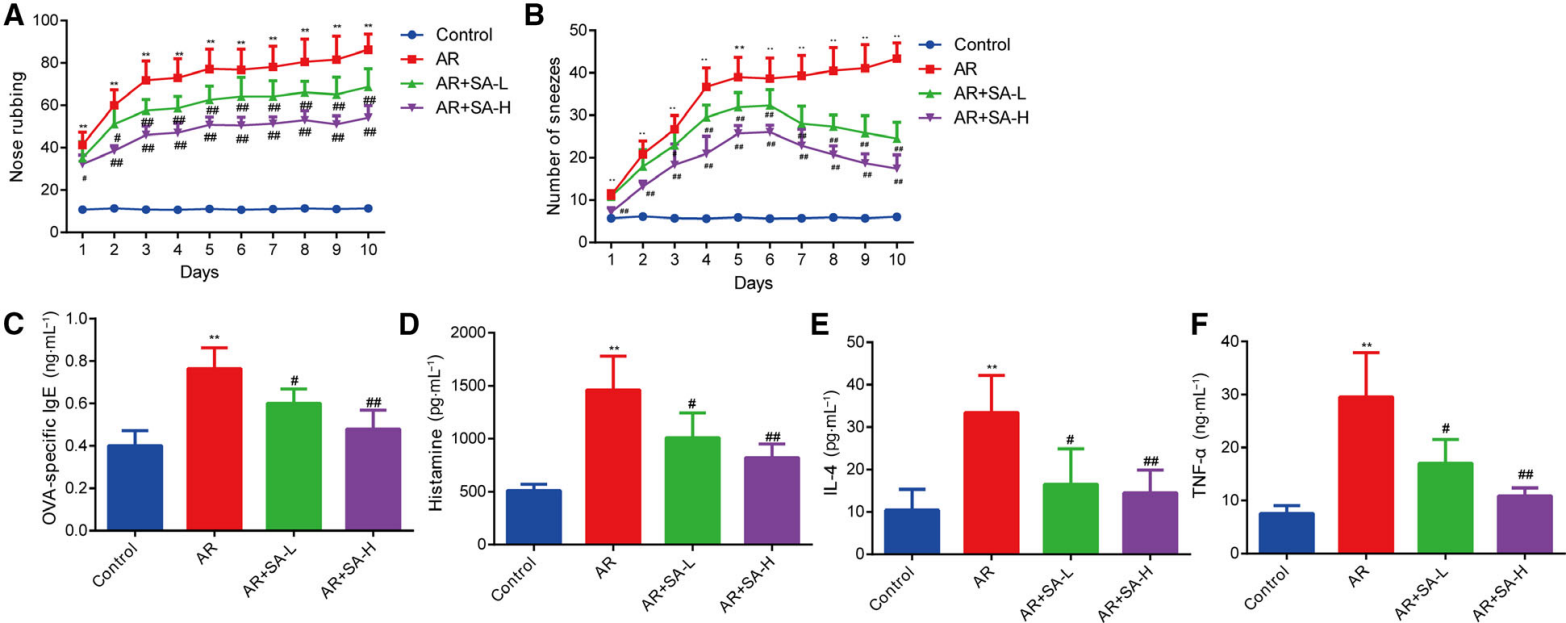
Salvinorin A inhibited OVA‐induced AR in mice. (A) Count of the amount of nasal rubbing within 10 min after intranasal OVA injection. (B) The number of sneezes within 10 min after intranasal OVA injection was counted. (C) Salvinorin A inhibited the level of OVA‐specific IgE in OVA‐induced AR in mice. (D) Salvinorin A inhibited the level of histamine in OVA‐induced AR in mice. (E) Salvinorin A suppressed the level of IL‐4 in OVA‐induced AR in mice. (F) Salvinorin A suppressed the level of TNF‐α in OVA‐induced AR in mice. **P < 0.01, in comparison with the control group; ^#^ *P* < 0.05, ^##^ *P* < 0.001, in comparison with the AR group. All data from three independent experiments are expressed as the mean ± SEM. The difference between different groups was analyzed by one‐way ANOVA, followed by Bonferroni’s *post hoc* test.

To further understand the effects of salvinorin A on AR, we examined the effects of salvinorin A in AR mouse models. Salvinorin A was administered intraperitoneally before an intranasal OVA challenge for 10 days; then the amount of nasal rubbing and sneezing was determined. The amount of rubbing and sneezing was significantly elevated in the AR group compared with the normal mouse group. In contrast, this effect was suppressed by administration of salvinorin A (Fig. 4A,B). To explore the function of salvinorin A in the allergic response *in vivo*, we determined the expression levels of both OVA‐specific IgE and histamine. Levels of OVA‐specific IgE and histamine were markedly down‐regulated in the groups that received salvinorin A compared with the AR mouse group (Fig. 4C,D). We also examined the IL‐4 and TNF‐α levels in serum. Similarly, IL‐4 and TNF‐α were markedly decreased by salvinorin A in AR mice compared with untreated AR mice (Fig. 4E,F).

### Salvinorin A repressed PI3K/Akt signaling in AR mice

**Fig. 5:**
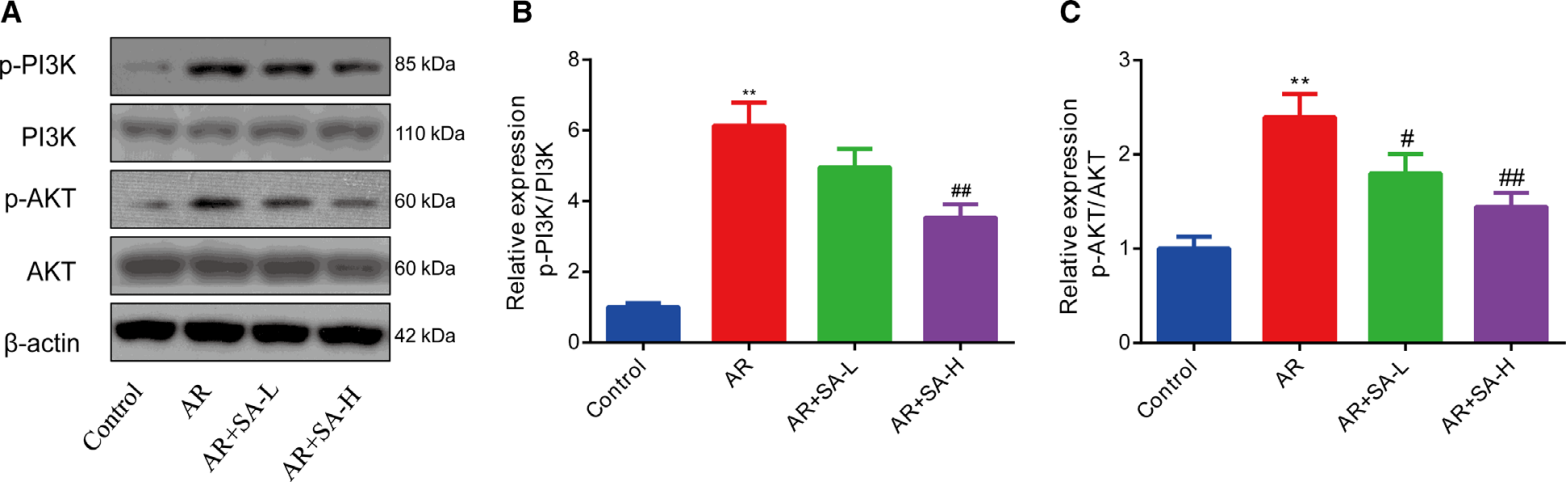
Salvinorin A repressed PI3K/Akt signaling in AR mice. (A) The relative PI3K (B) and Akt (C) protein expression levels in AR mice (*in vivo*) were determined by western blotting. **P < 0.01, in comparison with the control group; ^#^ *P* < 0.05, ^##^ *P* < 0.001, in comparison with the AR group. All data from three independent experiments are expressed as the mean ± SEM. The difference between different groups was analyzed by one‐way ANOVA, followed by Bonferroni's *post hoc* test.

We also investigated the mechanism underlying the inhibitory effects of salvinorin A in AR mice by examining the PI3K, p‐PI3K, Akt and p‐Akt protein expression levels. The phosphorylation of PI3K and Akt was clearly increased in AR mice, which was reversed by salvinorin A (Fig. 5A–C).

## Discussion

A number of studies in animal models have shown that salvinorin A exerts several potentially therapeutic pharmacological effects, classically acting on the central nervous system [15], as well as inhibiting LT production, LT‐related inflammatory parameters [16] and intestinal motility [17]. In particular, the suppression of airway hyperresponsiveness induced by OVA sensitization [14] attracted our attention. The antiallergic effect of salvinorin A has already been published in previous work [14]. The novelty of our findings was that salvinorin A inhibited OVA‐stimulated AR and RBL‐2H3 cell degranulation by regulating the PI3K/Akt signaling pathway.

The RBL‐2H3 cell line has been widely used to study IgE‐mediated mast cell activation as a result of strong expression of the IgE receptor FcεRI [18, 19, 20]. The mouse model of AR formed by OVA stimulation has been used in several studies [21, 22]. In this study, with the aid of cell‐based techniques and a mouse model, we constructed a DNP‐IgE/HSA‐sensitized RBL‐2H3 cell model to explore the function of salvinorin A in the activation of mast cells and OVA‐induced AR.

During IgE‐mediated mast cell activation, cells produce cytoplasmic particles containing proinflammatory and proallergenic mediators, which are released into the surrounding environment [23]. Degranulation is a marker of mast cell activation and the release of inflammatory mediators [24]. Cytoplasmic granules contain the degranulation indicators β‐hexosaminase and histamine [25]. IL‐4 is an indispensable molecule in allergic reactions, inducing homotypic conversion to IgE, up‐regulating adhesion molecules and promoting eosinophil migration [26, 27]. TNF‐α promotes inflammation caused by neutrophils and T cells, leukocyte infiltration, and chemotaxis, while mast cells activate TNF‐α secretion, which plays a key role in allergic reactions [28, 29]. Using *in vivo* and *in vitro* models, our study established that salvinorin A reduces β‐hexosaminidase, histamine, IL‐4 and TNF‐α levels, indicating that salvinorin A can alleviate AR by inhibiting inflammation and histamine release.

Coptisine effectively suppressed the phosphorylation of PI3K/Akt in the RBL‐2H3 cell line [18]. LV‐KCa3.1 short hairpin RNA intervention significantly reduced mast cell activity by blocking activation of the PI3K/AKT signaling pathway [30]. miR‐126 accelerated IgE‐mediated mast cell degranulation associated with the PI3K/Akt signaling pathway [31]. These results suggest that the PI3K/Akt signaling pathway is activated in AR. Thus, we also detected the PI3K/Akt signaling pathway after salvinorin A treatment and found that phosphorylation of PI3K/Akt was blocked by salvinorin A in both *in vivo* and *in vitro* models.

Antiallergic effects shown in the previous study seem to be complicated [14]. The author showed that salvinorin A up‐modulated IgE response, whereas Th2 cytokine production was suppressed. The conclusion was that salvinorin A inhibited the Th2 cytokine production, such as IL‐4, which was the same as our findings. The author explained that salvinorin A up‐modulated the IgE response and was coupled to a significant increase in bronchial hyperreactivity and pulmonary inflammation associated to pulmonary mast cell recruitment [32], which was contrary to our results. We have known that mast cells are activated by cross‐linking of allergen‐IgE bound to FcεRI on the cell surface, culminating in degranulation releasing mediators that activated PI3K and phosphorylation PI3K activated Akt [33]. Our results showed that salvinorin A down‐regulated the IgE response via the PI3K/Akt signing pathway. This result was consistent with a previous study that confirmed that coptisine treatment obviously improved nasal symptom scores and inhibited the elevation of serum IgE as a result of its antioxidant and anti‐inflammatory effects on AR [18]. In summary, this study provides evidence of salvinorin A inhibition of IgE‐mediated allergic reactions and OVA‐induced AR in mice, which has implications for development of anti‐AR drugs.

## Conclusions

Overall, this study provides evidence of salvinorin A inhibition of IgE‐mediated allergic reactions and OVA‐induced AR in mice and has implications for the development of anti‐AR drugs.

## Conflict of interest

The authors declare no conflict of interest.

## Author contributions

QS and TT performed the experiments and conducted data analysis. FX designed the study and revised the manuscript. QS wrote the first version of the manuscript.

## Data Availability

The analyzed datasets generated during the study are available from the corresponding author on reasonable request.
